# An investigation into blood microbiota and its potential association with Bacterial Chondronecrosis with Osteomyelitis (BCO) in Broilers

**DOI:** 10.1038/srep25882

**Published:** 2016-05-13

**Authors:** Rabindra K. Mandal, Tieshan Jiang, Adnan A. Al-Rubaye, Douglas D. Rhoads, Robert F. Wideman, Jiangchao Zhao, Igal Pevzner, Young Min Kwon

**Affiliations:** 1 Department of Poultry Science, University of Arkansas, Fayetteville, Arkansas, USA; 2 Cell and Molecular Biology Program, University of Arkansas, Fayetteville, AR, USA; 3 Department of Animal Science, University of Arkansas, Fayetteville, AR, USA; 4 Cobb-Vantress Inc., Siloam Springs, AR, USA

## Abstract

Bacterial chondronecrosis with osteomyelitis (BCO) is a common cause of lameness in commercial broiler chickens worldwide. BCO represents substantial production loss and welfare issues of chickens. The bacterial species or communities underlying BCO pathogenesis still remain to be fully characterized. To gain insights on blood microbiota in broilers and its potential association with BCO, blood samples collected from healthy (n = 240) and lame (n = 12) chickens were analyzed by deep sequencing of 16S RNA genes. The chicken blood microbiota were dominated by *Proteobacteria* (60.58% ± 0.65) followed by *Bactroidetes* (13.99% ± 0.29), *Firmicutes* (11.45% ± 0.51), *Actinobacteria* (10.21% ± 0.37) and *Cyanobacteria* (1.96% ± 0.21) that constituted 98.18% (± 0.22) of the whole phyla. The bacterial communities consist of 30–40 OTUs in the blood of broiler chickens, regardless of ages and other environmental or host conditions, and the blood microbiomes of BCO chickens were largely distinct from those of healthy chickens. In addition, Linear discriminant analysis (LDA) effect size (LEfSe) method revealed that *Staphylococcus*, *Granulicatella*, and *Microbacterium* were significantly enriched in BCO chickens as compared to healthy chickens. The results from this study have significant implications in understanding blood microbiota present in broiler chickens and its potential role in BCO pathogenesis.

Bacterial chondronecrosis with osteomyelitis (BCO, also known as femoral head necrosis) is a common cause of lameness in commercial broiler chickens worldwide. It was first recognized almost half century ago in 1972^1^. BCO represents substantial production loss and compromised welfare issues of chickens^2^. Although numerous bacterial species, including *Staphylococcus aureus*, *Escherichia coli*, *Salmonella* and *Enterococcus* have been isolated from the femurs or tibias of BCO birds^3^,^4^, the bacterial species or communities underlying BCO pathogenesis still remain to be fully characterized. Recently, we have conducted a comprehensive survey of bacterial communities in the leg bones of BCO chickens for the first time by high-throughput sequencing of 16S rRNA gene^4^. The existence of complex bacterial communities in the BCO bones supported by the study suggested that the bacterial species may transmit into the leg bones through blood stream after they are translocated from the intestinal tract or respiratory tract to the blood^5^. Subsequently, micro-trauma to poorly mineralized columns of cartilage cells in the proximal growth plates of leg bones is colonized by the bacteria. The bacterial colonization results in insufficient blood flow to growth plate, vascular occlusion, and structural limitations of the microvasculature, leading to BCO^5^.

Therefore, our better understanding of the bacterial cause of BCO necessitates characterization of microbiota potentially present in the blood of BCO birds. We also need to understand the magnitude and persistence of the bacteremic response and their correlation with BCO lameness in chickens. In humans, dysbioses of intestinal microbiota have been linked to a variety of disorders such as inflammatory bowel diseases, cancers, obesity, diabetes, chronic fatigue syndrome, bacterial vaginosis, autism, asthma, and infectious colitis among others^6^,^7^, and potential biomarkers associated with these conditions have been identified^8^. While blood in healthy organism was first believed to be a sterile environment, the presence of dormant and not-immediately-culturable forms of microbes in the blood has been well established^9^. Thus, it may suggest that certain perturbations in blood microbiota either resulting from translocation of bacteria from gut or respiratory tract due to changes in environments or host conditions can lead to disease status in the host^10^. A recent study indicates that plasm apolipoprotein A-I (APOA1) and its catabolic product may be used as a biomarkers for femur head separation (FHS)^11^, which is the initial stage of BCO^3^. Even though a number of studies have been done on bacterial microbiota in poultry, most studies are focused on gut microbiomes^4^,^12^,^13^,^14^,^15^,^16^,^17^,^18^,^19^,^20^,^21^ and only a limited number of studies analyzed microbiomes in other body sites, including bone^4^, trachea^22^, and feces^23^. Here in this study we have explored blood microbiota of healthy birds as well as BCO birds in an attempt to gain general characteristics of blood microbiota and to identify bacterial groups in blood that are uniquely associated or enriched with BCO lameness via 16S rRNA gene profiling by high-throughput sequencing.

## Materials and Methods

### Ethics Statement

All the animal work described in this study was approved by the Institutional Animal Care and Use Committee (IACUC) at the University of Arkansas, and all experiments were performed in accordance with the approved guidelines and regulations.

### Animal Experiment Design

The arrangement of 24 pens was made as shown in Supplementary Fig. 1. Briefly twelve pens were set up with clean wood shavings litter flooring and twelve pens were set up with flat wire flooring. There were 4 different groups of pens: Chicks started on litter and remained on litter for 56 days (L1–56). Chicks started on litter and remained on litter for 34 days (L1–34) and were transferred to wire floor on day 35 and remained on wire till day 56 (W35–56). Chicks started on wire and remained on wire for 56 days (W1–56). The W35–56 pens remained empty until day 34. Cobb 500 FF byproduct male chicks from the hatchery in Fayetteville, AR were wing-banded and placed on the day of hatch in the 16 pens (excluding the 8 pens for W35–56) at approximately 80 birds per pen, and were culled to 60 per pen on day 14. Feed and water were provided *ad libitum*. The starter diet was a commercial corn and soybean-meal based chick starter (crumbles), and on day 35 all birds were switched to a pelleted commercial corn and soybean-meal based finisher diet. The birds were grown rapidly throughout the experiment (23 hours of light, full feed, optimal temperature and ventilation conditions). For the wire flooring, tube and feeder were provided on one side of the pen and nipple waterers was positioned on the other side of the pen, thereby forcing the birds to traverse the length of the floor to eat and then drink as shown in Supplementary Fig. 2^3^,^24^. This wire flooring provides a reliable experimental model for triggering significantly elevated incidences of BCO in research flocks^3^. The broilers were grown until day 56, then representative numbers of chickens were humanely euthanized and necropsied to assess BCO lesions.

### Blood Sampling and DNA extraction

Blood samples were collected from 5 apparently healthy birds per pen on day 14, 41 and 49 of age for microbiota analysis, yielding 240 blood samples (5 birds per pen × 16 pens per sampling age × 3 sampling ages). Additional 12 blood samples were collected from birds that succumb to BCO (n = 12; 5 and 7 samples from 41 and 49 day of age, respectively) making a total of 252 samples to be analyzed for 16S rRNA profiling. Blood samples were collected aseptically from a wing vein using EDTA Vacutainers. One ml of blood sample was centrifuged (5,000 rpm for 5 min at room temperature) using a microcentrifuge, and 200 μl buffy coat was collected in the sterile laminar flow chamber and stored at −20 °C. Genomic DNA was extracted from the buffy coats using BiOstic® Bacteremia DNA Isolation Kit (MoBio) following manufacturer’s instruction. DNA samples were analyzed using Qubit 2.0 Fluorometer (Life Technologies) for quantity and purity, and stored at −20 °C.

### PCR Protocol for 16S rRNA Gene amplification

**Figure 1:**
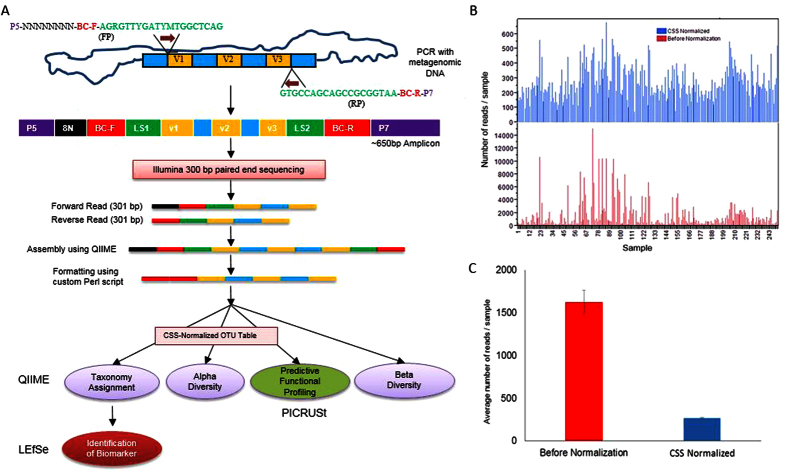
Schematic strategy for 16S rRNA sequencing. (**a**) Flow diagram representing library preparation, formatting reads acceptable to QIIME analysis which were further analyzed using LefSe and PICRUSt. *Normalized OTU table was used for PICRUST analysis. (FP = forward primer, RP = reverse primer, V = variable region of 16s RNA gene, P5 and P7 = Illumina sequencing primers, N = random nucleotide, BC-F = barcode of forward primer, BC-R = barcode of reverse primer, and LS = linker sequence). (**b**) Bar graph showing reads distribution of 252 samples before (red) and after normalization (blue) of OTU table. (**c**) Bar graph showing average number of reads per sample with standard error before (red) and after normalization (blue).

DNA isolated from blood samples were used as templates to amplify V1-V2-V3 regions using barcode-tagged universal primers 27F (5′-AGRGTTYGATYMTGGCTCAG-3′) and 533R (5′-TTACCGCGGCTGCTGGCAC-3′) to which Illumina adapter sequences were attached as shown in Fig. 1. The full set of primers used in this study are shown in Supplementary Table 1. The steps for decontamination of DNA and PCR were performed inside a sterile laminar flow chamber. For decontamination of DNA that potentially contaminate PCR reagents prior to PCR step, the reaction that consisted of 23.5 μl DNA- free H_2_O (Mo Bio Laboratories, Inc., Carlsbad, CA), 3 μl 10× buffer II, 0.5 μl AccuPrime™ Taq DNA Polymerase High Fidelity (Life Technolgies, Grand Island NY), 0.5 μl forward primer and reverse primer each (20 μM), 0.5 μl Dithiothreitol, 0.62 μl dsDNase (AcrticZymes Tromsø, Norway) in total 29.12 μl volume was prepared, and incubated at 37 °C for 30 min, followed by inactivation of dsDNase by heating at 60 °C for 20 min. Immediately, PCR was conducted with addition of 1 μl of genomic DNA into the reaction tube inside laminar flow chamber, making 30.12 μl final volume with following conditions: 94 °C for 1 minutes; 35 cycles of 94 °C sec for 30 sec, 58 °C for 30 sec, 68 °C for 30 sec; and 68 °C for 5 minutes for final extension. Ten μl of each PCR product (≈650 bp) was run on 1% agarose gel to estimate the band intensity of 16S rRNA gene amplicons. The intensities of the amplified 16S rRNA gene fragments were generally very low even after 35 cycles of PCR, because the genomic DNA samples contained relatively small amount bacterial DNA in the background of much larger amount of chicken DNA. If the amplicons were gel-purified from individual samples, the concentration would be too low to be used in the following steps. Therefore, we used the following strategy to combine the individual samples: all of the 252 samples were classified into 4 categories (1^st^, 2^nd^, 3^rd^ and 4^th^; 1^st^ being the strongest band and 4^th^ the faintest band) according to the relative band intensities. Then PCR products in the same categories were mixed together in the same volume within each category. The mixed DNA samples in these 4 categories were PAGE-purified using 4–20% polyacrylamide gel (Life Technologies, NY, USA). These 4 category DNA samples were then mixed together so that approximately the same quantity of DNA (≈1.53 ng/sample) from each of 252 samples would be included in the final mixed sample, based on the measurement with Qubit 2.0 Fluorometer. Twenty μl of the final mixed sample (19.4 ng/ul) was sent to Genomics Core Facility at the University of California at Riverside for Illumina sequencing using MiSeq with 301 cycles in paired-end mode.

### Data Analysis

The flowchart that outlines the analysis of 16S rRNA sequence reads is shown in Fig. 1a. Forward and reverse paired-end reads from Illumina sequencing were assembled using Quantitative Insights into Microbial Ecology, QIIME 1.9.1^25^, via join_paired_ends.py script using fastq-join method. Then barcode sequences of forward read (BC-F) and reverse read (BC-R) were joined and brought together (5′ end) with the assembled sequence (3′ end) after discarding 8 nucleotide random sequence and 27F and 533R primer sequences using custom Perl script, which produced fastq file compatible to downstream analysis using QIIME.

Reads were clustered using UCLUST^26^ and operational taxonomic units (OTU) picking with closed reference option was performed using 13_8 GreenGenes database release. OTU BIOM (biological observation matrix) table was normalized with QIIME (normalize_table.py) using cumulative sum scaling (CSS) method, which was then used for taxonomic assignments, alpha diversity estimation, biomarker identification, and prediction of metagenome functional content. Alpha diversity indices were compared using one-way Analysis of Variance (ANOVA) and *post hoc* analysis was done with Tukey-Kramer HSD method. Analysis of similarities (ANOSIM) between the groups of metadata was performed using unweighted UniFrac distance metric with QIIME (compare_categories.py). Additionally, principal coordinate analyses (PCoA) for beta diversity estimates were performed with QIIME (24) using weighted and unweighted UniFrac metrics at different even sampling depth.

Identification of biomarker was performed using linear discriminant analysis effect size (LEfSe)^27^. Metagenome functional contents of chicken blood microbiota were predicted with PICRUSt (Phylogenetic Investigation of Communities by Reconstruction of Unobserved States) using 16S rRNA gene sequences^28^. Bacterial network was visualized using Cytoscape 3.2.1^29^.

## Results

QIIME demultiplexing and quality filtering produced 4,153,965 assembled sequence reads ranging from 40 to 580 bp with median length of 469 bp, which indicates the presence of chimeric sequences due to aberrant PCR amplification or incorrect assembly. We have used the closed reference OTU picking method in this study, because we are exploring a new type of microbiome for which the community structure, diversity and membership are largely unknown. However, we also compared the results obtained using closed reference method to those by both open reference and de novo OTU picking methods (Supplementary Table 2). Although open reference and de novo OTU picking method produced higher number of OTUs (on avg. 196 and 218, respectively) as well as higher reads per sample, there were significantly large portions of the reads (on avg. 72.5 and 79.3%, respectively) that were not assigned to any taxonomic groups in the current 13_8 GreenGenes database. In addition, to remove any noise or chimeric sequences in the downstream analysis effectively, we preferred closed reference OTU picking over the other two methods.

Closed reference OTU table had mean sample depth of 1,621 reads per sample (± 139.07) as shown in Fig. 1b. OTU table was normalized using cumulative sum scaling (CSS) with QIIME (Fig. 1b, c). Although the mean reads of the samples were decreased by normalization more than 6 fold (266.55 per sample), it also reduced the standard error (±7.01). The CSS normalized OTU table was used for taxonomy assignment, alpha diversity estimates, LEfSe, and PICRUt analysis. Beta diversity analysis were performed at various sampling depth of reads and ANOSIM with 999 permutations using unweighted UniFrac metric at even sampling depth of 400 reads per sample.

### Taxonomy Assignment

**Figure 2:**
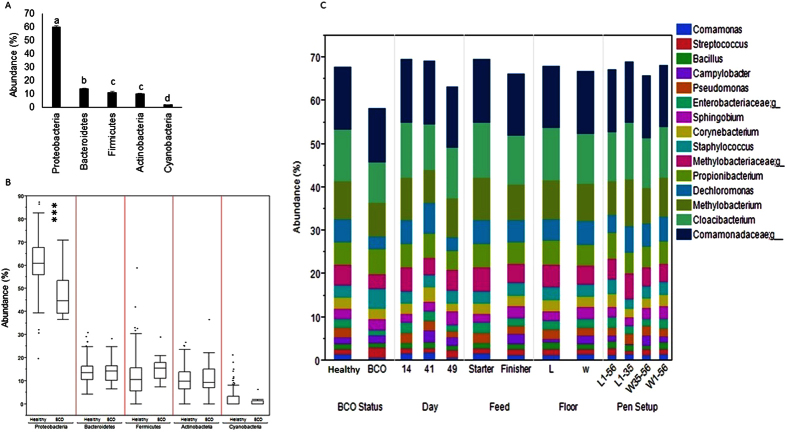
Distribution of taxa at different level. (**a**) Bar chart at phylum level with mean. Different letters above each bar indicate significant different (ANOVA, Tukey-Kramer test, p < 0.05). (**b**) Box plot showing outliers at phylum level (healthy and BCO birds). (**c**) Stacked area graph showing abundance of top 15 genus at Day (14, 41 vs 49 day of age), BCO status (healthy vs BCO), Floor (L = Litter vs W = Wire), Feed (Starter vs Finisher), and Pen Setup (L1–56, L1–35, W35–56 vs. W1–56) categories.

The chicken blood microbiota were dominated by *Proteobacteria* (60.58% ± 0.65) followed by *Bactroidetes* (13.99% ± 0.29), *Firmicutes* (11.45% ± 0.51), *Actinobacteria* (10.21% ± 0.37) and *Cyanobacteria* (1.96% ± 0.21) that constituted 98.18% (± 0.22) of the whole phyla. Other minor abundant phyla included *Palncotmytcete*, *Acidobacteria*, *Chloroflexi*, *Fusobacteria*, *Gemmatimonadetes*, *Verrucomicobia*, *WS4* and *Thermi*. The abundance levels of *Firmicutes* and *Actinobacteria* did not show significant difference while the abundance levels of other phyla were significantly different with each other (Tukey-Kramer test, p < 0.05) (Fig. 2a). The abundance of *Proteobacteria* was significantly lower in BCO birds as compared to healthy birds (t-test, p < 0.0001). Although there was slight increase in abundance of *Firmicutes* in BCO chickens than healthy birds, the difference was not significant (t-test, p > 0.05). Additionally, there was no significant difference between BCO and healthy birds in the abundance of other major phyla (t-test, p > 0.05) as shown in Fig. 2b. There was no BCO bird among the day 14 samples, because BCO occurs mostly after 35 days of age although it has been observed in birds ranging from 14 to 70 days of age^1^. The top 15 abundant genera, including 3 taxonomic groups whose genera were not identified at 97% sequence similarity constituted more than 67.3% of the whole blood microbiota. These genera included *Cloacibacterium* (11.7%), *Methylobacterium* (8.7%), *Dechloromonas* (5.3%), *Propionibacterium* (5.1%), *Staphylococcus* (2.9%), *Sphingobium* (2.5%), *Corynebacterium* (2.5%), *Pseudomonas* (2%), *Campylobacter* (1.6%), *Streptococcus* (1.3%), *Bacillus* (1.3%) and *Comamonas* (1.3%), and three families were *Comamonadaceae* (14%), *Methylobacteriaceae* (4.7%) and *Enterobacteriaceae* (2%). The relative abundance of the genera were compared between the groups according to different criteria as shown in Fig. 2c. Although *Staphylococcus* was higher in BCO birds as compared to control, but it was not significant (t-test, p > 0.05). Interestingly, there was no significant difference in the relative abundance of *Campylobacter* between BCO and healthy birds (t-test, p > 0.05), however significant difference was observed between the groups of other criteria (Day, Feed, and Floor). The same trend was observed with *Methylobacterium*. Also the relative abundance levels of most of the major genera were significantly higher at 41 day (p < 0.05) than 14 and 49 days. Additionally, *Pseudomonas* was significantly higher in healthy chickens (t-test, p < 0.05) than BCO chickens.

### Alpha Diversity

**Figure 3:**
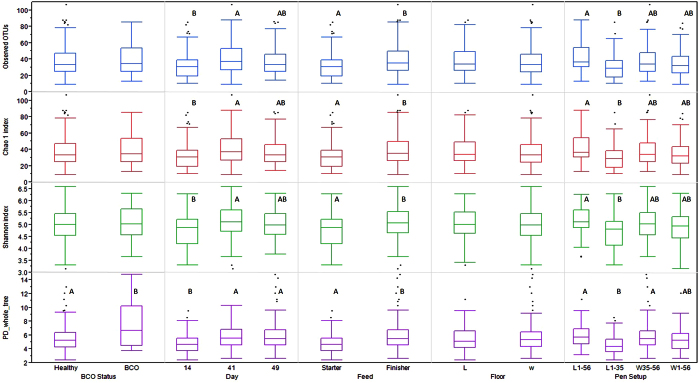
Alpha diversity index of CSS normalized blood microbiota OTU table. Box plot of experimental category with outlier. The boxes display median, 25th, and 75th percentiles with outliers outside lower and upper extremes. Boxes with different letters are significantly different (p < 0.05).

Alpha diversity is the measurement of the diversity within a community, and there are different metrics that have been devised to measure alpha diversity with emphasis on different aspects of the community structure. Overall the chicken blood microbiota had an average of 37.21 (±1.13) OTUs (min = 9; max = 107) at 97% sequence similarity (Fig. 3; Supplementary Table 3). A significant contrast was seen in alpha diversity of BCO vs healthy chicken with PD whole Tree index (t-test, p < 0.05), but not with other metrics. Similarly, 41 day old chickens showed significantly different alpha diversity as compared to 14 day old chickens, but there was no difference between 14 and 49 days with all metrics used in this study (Tukey-Kramer test, p > 0.05). Moreover, the chickens on starter feed vs finisher feed showed significant difference in alpha diversity (t-test, p < 0.05). The overall litter vs wire flooring showed no significant difference in alpha diversity (t-test, p > 0.05) (Fig. 3) which is in concordance with our previous study^4^.

### Beta Diversity

**Figure 4:**
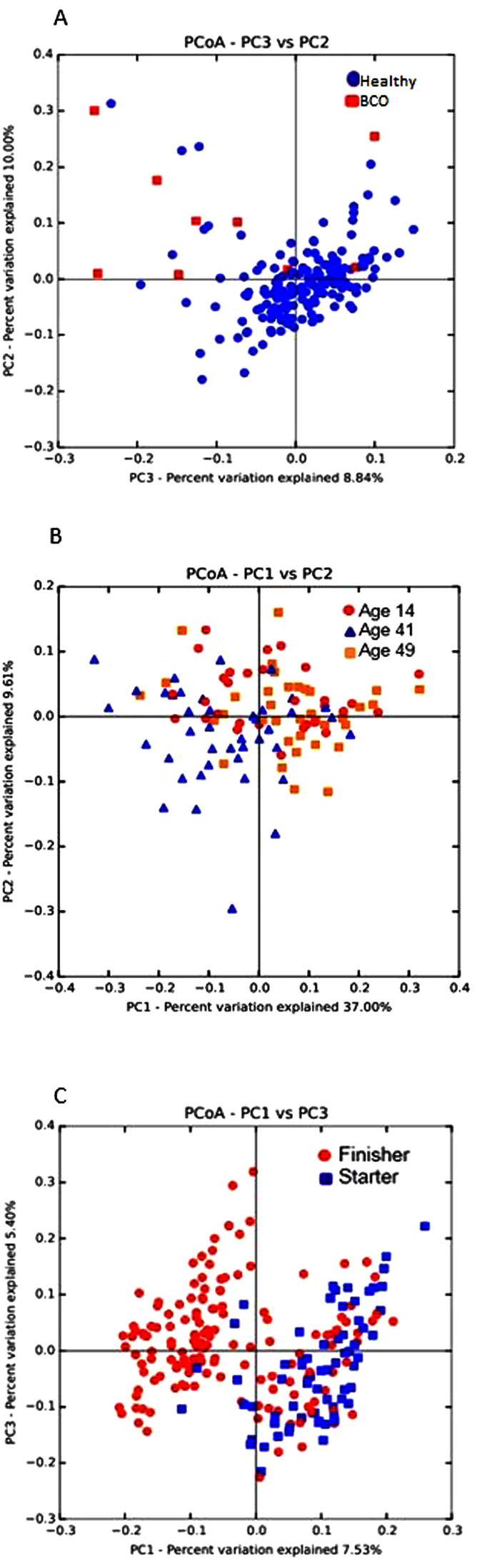
Principal Coordinate Analysis (PCoA) plot of blood microbiomes of chickens. (**a**) PCoA plot of healthy (n = 171) vs BCO (n = 9) chickens using weighted UniFrac metric at even sampling depth of 400 read per sample. (**b**) PCoA plot of 14 day (n = 30), 41 day (n = 41) vs 49 day (n = 36) old chickens with weighted UniFrac metric at even sampling depth of 1,000 reads per sample. (**c**) PCoA plot of starter (n = 67) vs finisher (n = 154) diet fed chickens using unweighted UniFrac metric at even sampling depth of 200 read per sample.

Analysis of similarities (ANOSIM) based on unweighted UniFrac metric showed that BCO vs healthy chickens (R = 0.4402, p = 0.001) showed significant difference in the bacterial community structure. However, bacterial communities were not significantly different among the groups according to other criteria, including feed, floor, and pen setup (Table 1). R value equal to 1 shows that the samples are completely different, while 0 means that they are identical. It is important to note that there was significant difference in alpha diversity between BCO vs healthy birds (with PD whole tree index), and also among different age groups (Fig. 3). Principal coordinate analysis (PCoA) plot based on weighted Unifrac distance shows the distinct overall difference between BCO vs healthy chickens in the community structure (Fig. 4a). The bacterial communities of day 41 were also distinctively different from those of day 14 and 49 (Fig. 4b). This is in agreement with our previous observation that the alpha diversity indices of the communities of day 41 were significantly higher as compared to day 14 and 49. In addition, PCoA plot showed distinct bacterial communities between the chickens fed with starter and finisher feed (Fig. 4c), although ANOSIM was not able to capture this significant feature (Table 1).

**Figure 5:**
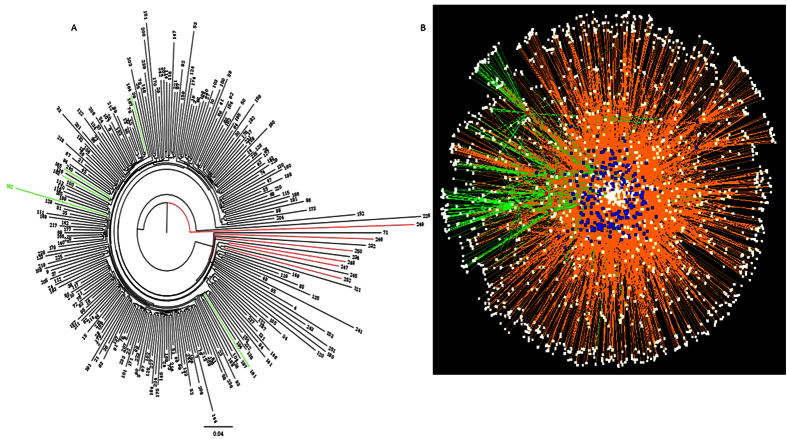
Clustering of chickens based on blood microbiota. (**a**) Hierarchical clustering of healthy vs BCO chickens based on blood microbiota. Phylogenetic tree was generated using FigTree software V1.3.1 with weighted UniFrac metric using the data before normalization. Green and red lines represent BCO chickens of 41 and 49 day old chickens, respectively. Black lines are healthy chickens. Number at the end of line shows the ID of chicken sample. (**b**) Bacterial network of chicken blood microbiota produced using Cytoscape software V3.2.1. Red, blue and white nodes represent BCO chickens, healthy chickens and OTUs, respectively. Green edge is the network of BCO chickens and orange edge is that of healthy chickens.

Significant difference in bacterial communities of healthy vs BCO chickens was also illustrated by hierarchical clustering. BCO chickens of 49 day old chickens were clustered distinctively from the healthy chickens. However, 41 day old BCO chickens did not show any pattern of distinctive clustering (Fig. 5a). Network analysis between chickens (252 samples) and OTUs (bacterial species) showed a certain degree of distinction in the interaction patterns between BCO and healthy chickens (Fig. 5b), suggesting dissimilar bacterial communities.

**Figure 6:**
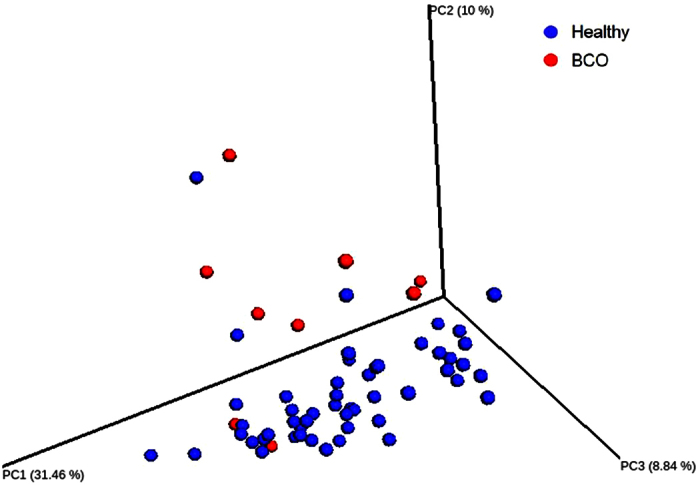
Principal Coordinate Analysis (PCoA) plot of blood microbiomes of a selected subset of chickens. PCoA plot of healthy (n = 51) vs BCO (n = 9) chickens using weighted UniFrac metric at even sampling depth of 400 read per sample.

We noticed all of the BCO birds were from only the two age groups (day 41 and 49) and two pen set up groups (W35–56 and W1–56). Therefore, we performed an additional beta diversity analysis using a subset of 77 samples that belong to those age and pen set up groups, including 65 healthy birds and 12 BCO birds. ANOSIM analysis based on weighted UniFrac metric indicated greater separation between BCO and healthy chickens (R = 0.5293, p = 0.001) as compared to that with the entire data set (n = 252). The PCoA plot shown in Fig. 6 also supports the result of the ANOSIM analysis.

### Biomarkers of BCO

**Figure 7:**
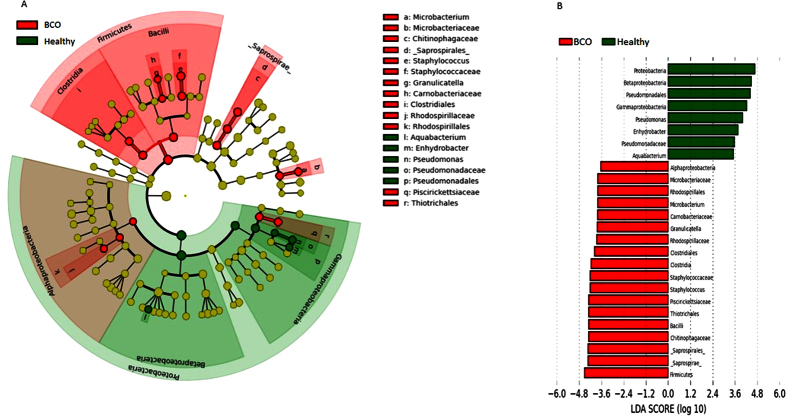
Differentially abundant taxa identified using LEfSe analysis using blood microbiota of chickens with relative abundance ≥0.1. (**a**) Taxonomic cladogram produced from LEfSe analysis. Red and green shows taxa enriched in BCO and healthy chickens, respectively. Brightness is proportional to the abundance of taxon. (**b**) Taxa enriched in BCO chickens are shown in red with negative LDA score and healthy chickens in green with positive LDA score (>3.5 in both cases). Taxon between two underscore is proposed name of GreenGene database.

The taxonomic groups that are differentially abundant between healthy vs BCO chickens were identified using linear discriminant analysis effect size (LEfSe) with α = 0.05, LDA score of at least 2, and relative abundance greater than 0.1. A total of 26 features had significantly different abundance between healthy and BCO chickens. At a genus level, blood microbiota of BCO chickens were differentially enriched with genera *Staphylococcus*, *Granulicatella*, and *Microbacterium*, whereas healthy chickens were enriched with *Pseudomonas*, *Enhydrobacter* and *Aquabacterium* (Fig. 7b). We also observed that the phylum *Firmicutes* was enriched in BCO chickens. Similarly, *Alphaproteobacteria* was highly enriched in BCO chickens while *Betaproteobacteria* and *Gammaproteobacteria* in healthy chickens at class level (Fig. 7b).

### Predicted Functional Genetic Capacity of the Chicken Blood Microbiomes

**Figure 8:**
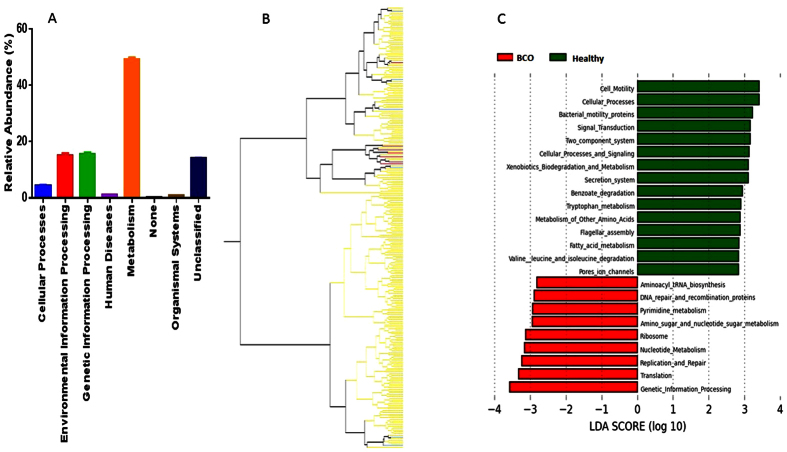
Predicted functional composition of chicken blood microbiota using PICRUSt. (**a**) Relative abundance of KEGG COG categories at level 1 in chicken blood microbiomes. (**b**) Relative abundance (≥0.5) of KEGG pathway at level 3 categories of functional pathways generated using JMP software. (**c**) Differentially abundant features (KEGG COG categories, relative abundance ≥0.5) produced using LEfSe with LDA score ≥2.8.

Normalized closed reference OTU matrix was used to predict the genetic potentials of the blood microbiome metagenomes using phylogenetic investigation of communities by reconstruction of unobserved states (PICRUSt)^28^ on web-based platform Galaxy (http://huttenhower.sph.harvard.edu/galaxy). Relative abundance levels of the functional pathways potentially encoded by the metagenomes were estimated using PICRUSt based on Kyoto Encyclopedia of Genes and Genomes (KEGG) cluster of orthologous groups (COGs). Predicted metagenomes of the blood microbiota were highly related to Metabolism (49.15%) followed by similar level of Genetic information processing and Environmental information processing (15%). Abundance of genes related to Human disease was 1.14%; Organismal systems 0.81%; none 0.20% and unclassified metagenome was 13.93% (Fig. 8a). Hierarchical clustering of 252 chicken samples based on the pathways with abundance ≥0.5% at level 3 categories of the functional pathways showed that 6 out of 7 BCO chickens of 49 days old were clustered together showing similar metagenomic genetic potential among BCO chickens. However, BCO chickens of 41 day old age were not clustered, indicating diverse blood microbiota at that stage of chickens (Fig. 8b). Metagenomic genetic pathways differentially enriched in the BCO chickens were related mostly to DNA replication, Repair or metabolism as identified using LEfSe. These included Genetic information processing, Translation, Replication and repair, Nucleotide metabolism, DNA repair and recombination proteins among others. However, predicted features enriched in healthy chicken blood microbiota were related to level 1 categories Cellular process and signaling and Environmental information processing. Additionally, metabolism pathways associated with fatty acid, tryptophan and xenobiotic were differentially abundant in the blood microbiomes in healthy chickens (Fig. 8c).

## Discussion

This is the first comprehensive study analyzing bacterial microbiomes that exist in the blood of non-human vertebrate animals. There has been a burgeoning interest in recent years to characterize microbiota associated with different tissues of body under different health conditions. The body parts once thought to be sterile, such as blood^30^, stomach^31^, bladder^32^, lungs^33^, bones, joints^4^ and breast^34^ have their indigenous microbiota. Tissue microbiota dysbiosis has been linked to various diseases, including cardiovascular disease^10^, diabetes^35^, non-alcoholic fatty liver disease (NASHD)^36^, inflammatory bowel disease (IBD)^37^, psoriasis^38^, obesity^39^, childhood-onset asthma^39^, functional bowel disease^40^, and colorectal carcinoma^41^. Here, we have investigated into the blood microbiota of chickens with the objective of identifying potential bacterial biomarkers associated with BCO.

We found the most abundant phylum was *Proteobacteria* followed by *Bacteroidetes* in chicken blood microbiota (both healthy and BCO chickens), which are in concordant with human blood microbiota study conducted by Amar *et al.*^30^. Moreover, *Firmicutes*, *Actinobacteria* and *Cyanobacteria* were other abundant phyla in both studies. However, the abundance of *Proteobacteria* in our study was lower (60.5%) than that in human blood microbiome (85–90%). It is important to note that most abundant phylum in chicken gut is *Firmicutes* followed by two minor phyla, *Proteobacteria* and *Bacterioidetes*. Other low abundant phyla in chicken gut include *Actinobacteria*, *Tenericutes*, *Cyanobacteria* and *Fusobacteria*^42^. Because of the discrepancy in the abundance of phyla in blood and gut, it is believed that blood microbiota may not be the result of bacterial cells passively present in blood after translocation from the gut. It may suggest that blood may harbor indigenous microbiota selected and stably maintained in that unique environment. Therefore, we might argue that the BCO could be due to blood microbiota dysbiosis^43^. LEfSe analysis in this study showed a significant increase of phylum *Firmicutes* (most abundant phylum in chicken gut) in BCO birds as compared to healthy birds, which might reflect the outcome of increased leakage of gut microbiota into blood (caused by the stress from wire flooring), causing blood microbiota dysbiosis in BCO chickens.

The result of data analysis in this study showed the existence of bacterial communities that consist of 30 to 40 OTUs in the blood of broiler chickens, regardless of ages and other environmental or host conditions. The beta diversity analysis (Figs 4a and 6), hierarchical clustering analysis (Fig. 5a), and bacterial network analysis (Fig. 5b) based on blood microbiota and hierarchical clustering based on predicted metagenome of blood microbiota (Fig. 8b) suggested that the bacterial communities in the blood of BCO birds are distinctive from those in healthy birds, suggesting the presence of certain selective pressures contributing to the shift in the blood microbiomes in BCO birds. However, only PD whole tree showed significant difference in alpha diversity between BCO and healthy birds, which is in agreement with phylogenetically distant blood microbiota in BCO birds as shown in beta diversity analysis (Figs 4a and 6).

In our unpublished companion study, the blood samples from the same flock were directly plated on rich agar media. The result showed that colony counts at day 49 were consistently higher on wire floors (L35W and W56) as compared to litter (L56), indicating stress on wire floor promoted bacteremia probably through bacterial translocation across the gut epithelium. In addition, the colony counts at day 49 were also consistently higher with lame birds as compared to healthy birds. These results indicate strong correlation among the stress from wire flooring, the severity of bacteremia, and BCO lameness.

One practical application of this study was to identify bacterial biomarkers that could be used to identify the individual broiler chickens at earlier ages that are prone to BCO development in later ages. The data analysis in this study indeed identified taxonomic groups at different levels that are significantly enriched in BCO birds as compared to healthy birds. Interestingly, the genus *Staphylococcus* is one of the 18 features (including 3 genera) significantly enriched in BCO samples, which highlights the importance of this genus that has been frequently isolated from BCO lesions^3^,^4^,^5^. Recently Al-Rubaye *et al.*^44^ reported that the challenge of broilers with *S.* *agnetis*, which was most frequently isolated *Staphyloccous* species from BCO legions in their study, significantly increased lameness from 10 (Control) to 40%, while the challenge with another isolate *Enterococcus faecalis* decreased the lameness incidence. It may signify the potential importance of *S.* *agnetis* as the causative agent of BCO pathogenesis in broiler chickens, although our 16S rRNA gene profiling data failed to provide meaningful information on *Staphylococcus* species due to the limited resolution of taxonomic assignment.

In this study, however, BCO was detected only in the birds of 41 and 49 days of age and thus the bacterial biomarkers has a limited value for early diagnosis of BCO-prone birds. This aspect should be considered carefully in the experimental design for the future studies to allow identification of potential bacterial biomarkers in blood samples of young birds that are predictive of BCO development in older ages.

The blood microbiomes analyzed in this study have significant implications on the health status of broilers chickens, including BCO pathogenesis as demonstrated in this study as well as other disease or stress conditions of broiler chickens.

## Additional Information

**How to cite this article**: Mandal, R. K. *et al.* An investigation into blood microbiota and its potential association with Bacterial Chondronecrosis with Osteomyelitis (BCO) in Broilers. *Sci. Rep.* **6**, 25882; doi: 10.1038/srep25882 (2016).

## Supplementary Material

Supplementary Information

## Figures and Tables

**Table 1:** Analysis of similarities (ANOSIM) using unweighted UniFrac metric at even sampling depth of 400 reads per sample.

Category	No. of sub Category	R-value	p-value
BCO status	Healthy vs. BCO	0.4402	0.001*
Age	14, 41 and 49	0.1245	0.001*
Floor	Litter vs. Wire	−0.0220	0.896
Feed	Starter vs. Finisher	−0.0010	0.507
Pen setup	L1–56, L1–34, W35–56 vs W1–56	0.0157	0.151

^*^Significantly different (p < 0.05).
