# Supplementary material for: An investigation into blood microbiota and its potential association with Bacterial Chondronecrosis with Osteomyelitis (BCO) in Broilers
Source: Sci Rep. 2016 May 13;6:25882. doi: 10.1038/srep25882 (PMC4865835; doi:10.1038/srep25882)
Supplement: Supplementary Information [file srep25882-s1.doc]

**An investigation into blood microbiota and its potential association with Bacterial Chondronecrosis with Osteomyelitis (BCO) in Broilers**

Rabindra K. Mandal^1^, Tieshan Jiang^1^, Adnan A. Alrubaye^2^, Douglas D. Rhoads^2^, Robert F. Wideman Jr.^1^, Jiangchao Zhao^3^, Igal Pevzner^4^ and Young Min Kwon^1, 2 *^

**Supplementary Information**

**Supplementary Figure 1.** Arrangement of pens in A364 East: Litter = wood shavings litter; Wire = flat wire flooring. L1-56: birds remain on litter for 8 weeks; L1-35: birds remain on litter until day 35; W1-56: birds remain on wire for 8 weeks; W35-56: birds are transferred from litter to wire on day 35.

**Supplementary Figure 2.** Details of a 10 ft. x 10 ft. pen with flat wire panel flooring.

**Supplementary Table 1. List of Forward and Reverse Primers used in this study.**

| **Name** | **BC-F** | **Forward primers** |
| --- | --- | --- |
| V123F01 | TGAC | AATGATACGGCGACCACCGAGATCTACACTCTTTCCCTACACGACGCTCTTCCGATCT-NNNNNNNN-tgac-AGRGTTYGATYMTGGCTCAG |
| V123F02 | AGAG | AATGATACGGCGACCACCGAGATCTACACTCTTTCCCTACACGACGCTCTTCCGATCT-NNNNNNNN-agag-AGRGTTYGATYMTGGCTCAG |
| V123F03 | ATGA | AATGATACGGCGACCACCGAGATCTACACTCTTTCCCTACACGACGCTCTTCCGATCT-NNNNNNNN-atga-AGRGTTYGATYMTGGCTCAG |
| V123F04 | TGCA | AATGATACGGCGACCACCGAGATCTACACTCTTTCCCTACACGACGCTCTTCCGATCT-NNNNNNNN-tgca-AGRGTTYGATYMTGGCTCAG |
| V123F05 | GCAT | AATGATACGGCGACCACCGAGATCTACACTCTTTCCCTACACGACGCTCTTCCGATCT-NNNNNNNN-gcat-AGRGTTYGATYMTGGCTCAG |
| V123F06 | CGAT | AATGATACGGCGACCACCGAGATCTACACTCTTTCCCTACACGACGCTCTTCCGATCT-NNNNNNNN-cgat-AGRGTTYGATYMTGGCTCAG |
| V123F07 | CTAG | AATGATACGGCGACCACCGAGATCTACACTCTTTCCCTACACGACGCTCTTCCGATCT-NNNNNNNN-ctag-AGRGTTYGATYMTGGCTCAG |
| V123F08 | GATC | AATGATACGGCGACCACCGAGATCTACACTCTTTCCCTACACGACGCTCTTCCGATCT-NNNNNNNN-gatc-AGRGTTYGATYMTGGCTCAG |
| V123F09 | TAGC | AATGATACGGCGACCACCGAGATCTACACTCTTTCCCTACACGACGCTCTTCCGATCT-NNNNNNNN-tagc-AGRGTTYGATYMTGGCTCAG |
| V123F10 | GACT | AATGATACGGCGACCACCGAGATCTACACTCTTTCCCTACACGACGCTCTTCCGATCT-NNNNNNNN-gact-AGRGTTYGATYMTGGCTCAG |
| V123F11 | ATGC | AATGATACGGCGACCACCGAGATCTACACTCTTTCCCTACACGACGCTCTTCCGATCT-NNNNNNNN-atgc-AGRGTTYGATYMTGGCTCAG |
| V123F12 | ACGT | AATGATACGGCGACCACCGAGATCTACACTCTTTCCCTACACGACGCTCTTCCGATCT-NNNNNNNN-acgt-AGRGTTYGATYMTGGCTCAG |
| V123F13 | TCGA | AATGATACGGCGACCACCGAGATCTACACTCTTTCCCTACACGACGCTCTTCCGATCT-NNNNNNNN-tcga-AGRGTTYGATYMTGGCTCAG |
| V123F14 | GTAC | AATGATACGGCGACCACCGAGATCTACACTCTTTCCCTACACGACGCTCTTCCGATCT-NNNNNNNN-gtac-AGRGTTYGATYMTGGCTCAG |
| V123F15 | CATG | AATGATACGGCGACCACCGAGATCTACACTCTTTCCCTACACGACGCTCTTCCGATCT-NNNNNNNN-catg-AGRGTTYGATYMTGGCTCAG |
| V123F16 | CGTA | AATGATACGGCGACCACCGAGATCTACACTCTTTCCCTACACGACGCTCTTCCGATCT-NNNNNNNN-cgta-AGRGTTYGATYMTGGCTCAG |
| V123F17 | CTGA | AATGATACGGCGACCACCGAGATCTACACTCTTTCCCTACACGACGCTCTTCCGATCT-NNNNNNNN-ctga-AGRGTTYGATYMTGGCTCAG |
| V123F18 | CAGC | AATGATACGGCGACCACCGAGATCTACACTCTTTCCCTACACGACGCTCTTCCGATCT-NNNNNNNN-cagc-AGRGTTYGATYMTGGCTCAG |
|  |  |  |
| **Name** | **BC-R** | **Reverse Primers** |
| V123R01 | TGAC | CAAGCAGAAGACGGCATACGAGATGTGACTGGAGTTCAGACGTGTGCTCTTCCGATCT-tgac-TTACCGCGGCTGCTGGCAC |
| V123R02 | AGAG | CAAGCAGAAGACGGCATACGAGATGTGACTGGAGTTCAGACGTGTGCTCTTCCGATCT-agag-TTACCGCGGCTGCTGGCAC |
| V123R03 | ATGA | CAAGCAGAAGACGGCATACGAGATGTGACTGGAGTTCAGACGTGTGCTCTTCCGATCT-atga-TTACCGCGGCTGCTGGCAC |
| V123R04 | TGCA | CAAGCAGAAGACGGCATACGAGATGTGACTGGAGTTCAGACGTGTGCTCTTCCGATCT-tgca-TTACCGCGGCTGCTGGCAC |
| V123R05 | GCAT | CAAGCAGAAGACGGCATACGAGATGTGACTGGAGTTCAGACGTGTGCTCTTCCGATCT-gcat-TTACCGCGGCTGCTGGCAC |
| V123R06 | CGAT | CAAGCAGAAGACGGCATACGAGATGTGACTGGAGTTCAGACGTGTGCTCTTCCGATCT-cgat-TTACCGCGGCTGCTGGCAC |
| V123R07 | CTAG | CAAGCAGAAGACGGCATACGAGATGTGACTGGAGTTCAGACGTGTGCTCTTCCGATCT-ctag-TTACCGCGGCTGCTGGCAC |
| V123R08 | GATC | CAAGCAGAAGACGGCATACGAGATGTGACTGGAGTTCAGACGTGTGCTCTTCCGATCT-gatc-TTACCGCGGCTGCTGGCAC |
| V123R09 | TAGC | CAAGCAGAAGACGGCATACGAGATGTGACTGGAGTTCAGACGTGTGCTCTTCCGATCT-tagc-TTACCGCGGCTGCTGGCAC |
| V123R10 | GACT | CAAGCAGAAGACGGCATACGAGATGTGACTGGAGTTCAGACGTGTGCTCTTCCGATCT-gact-TTACCGCGGCTGCTGGCAC |
| V123R11 | ATGC | CAAGCAGAAGACGGCATACGAGATGTGACTGGAGTTCAGACGTGTGCTCTTCCGATCT-atgc-TTACCGCGGCTGCTGGCAC |
| V123R12 | ACGT | CAAGCAGAAGACGGCATACGAGATGTGACTGGAGTTCAGACGTGTGCTCTTCCGATCT-acgt-TTACCGCGGCTGCTGGCAC |
| V123R13 | TCGA | CAAGCAGAAGACGGCATACGAGATGTGACTGGAGTTCAGACGTGTGCTCTTCCGATCT-tcga-TTACCGCGGCTGCTGGCAC |
| V123R14 | GTAC | CAAGCAGAAGACGGCATACGAGATGTGACTGGAGTTCAGACGTGTGCTCTTCCGATCT-gtac-TTACCGCGGCTGCTGGCAC |
| V123R15 | CATG | CAAGCAGAAGACGGCATACGAGATGTGACTGGAGTTCAGACGTGTGCTCTTCCGATCT-catg-TTACCGCGGCTGCTGGCAC |
| V123R16 | CGTA | CAAGCAGAAGACGGCATACGAGATGTGACTGGAGTTCAGACGTGTGCTCTTCCGATCT-cgta-TTACCGCGGCTGCTGGCAC |

**Supplementary Table 2.** Comparison of CSS normalized OTU tables of 3 different OTU picking methods

OTU Picking Method Avg. reads/sample±SE Avg. OTU±SE Min reads Max Reads Unassigned

Closed reference 266±7.01 37.20±1.13 73.93 678.51 0.00

Open reference 933.52±23.54 196.00±6.64 122.23 2684.34 72.5±0.68

De novo 999.25±26.37 218.92±8.86 120.81 3330.62 79.3±0.61

*Unassigned Taxonomy % (Mean±SE); Min=Minimum; Max=Maximum

**Supplementary Table 3.** Alpha Diversity estimation using CSS normalized closed OTU matrix

Category Sub-category Alpha-diversity Metrics (±SE)

(No. of sample) PD whole Tree Shannon Chao 1 No. of OTUs

BCO Status Normal (240) 5.42(±0.11) 4.97(±0.04) 37.10(±1.15) 37.10(±1.15)

BCO (12) 7.63(±1.11) 5.03(±0.23) 39.41((±6.15) 39.41((±6.15)

Age 14(80) 4.80(±0.17) 4.80(±0.08) 33.34(±1.94) 33.34(±1.94)

41(85) 5.73(±0.18) 5.10(±0.07) 40.72(±2.05) 40.72(±2.05)

49(87) 5.99(±0.26) 5.00(±0.07) 37.33(±1.82) 37.33(±1.82)

Feed Starter (80) 4.81(±0.17) 4.80(±0.08) 33.34(±1.94) 33.34(±1.94)

Finisher (172) 5.86(±0.16) 5.05(±0.05) 39.01(±1.37) 39.01(±1.37)

Floor Litter (100) 5.34(±0.17) 4.99(±0.07) 37.79(±1.77) 37.79(±1.77)

Wire (152) 5.65(±0.17) 4.95(±0.05) 36.82(±1.47) 36.82(±1.47)

Pen setup L1-35 (40) 4.58(±0.25) 4.70(±0.72) 31.25(±2.67) 31.25(±2.67)

L1-56 (60) 5.85(±0.20) 5.18(±0.07) 42.15(±2.19) 42.15(±2.19)

W1-56 (63) 5.40(±0.24) 4.92(±0.09) 36.03(±2.24) 36.03(±2.24)

W35-56 5.82(±0.24) 4.99(±0.07) 37.38(±1.95) 37.38(±1.95)

Overall Mean 5.53(±0.12) 4.97(±0.04) 37.21(±1.13) 37.21(±1.13)
